# Ameliorative Effect of Rice Husk Methanol Extract on Liver and Kidney Toxicities Induced by Subchronic Codeine Administration

**DOI:** 10.1155/2023/3940759

**Published:** 2023-03-04

**Authors:** Chinonso U. Nnadiukwu, Eugene N. Onyeike, Catherine C. Ikewuchi, Kingsley C. Patrick-Iwuanyanwu

**Affiliations:** ^1^Africa Centre of Excellence in Public Health and Toxicological Research (PUTOR), University of Port Harcourt, PMB, 5323 Port Harcourt, Rivers State, Nigeria; ^2^Department of Biochemistry, Faculty of Sciences, University of Port Harcourt, Rivers State, Nigeria

## Abstract

*Background and Objective*. Rice husk remains a key by-product of rice milling generated in significant amount. Accumulated evidence indicates that rice husk contains numerous bioactive compounds; however, its application is limited. This study was designed to introduce an in vivo application of rice husk extract, against opioid-induced liver and kidney injuries. Codeine was considered a psychotic inducer in this study due to its global alarming misuse recently. The hepatorenal ameliorative proclivity of rice husk extract against codeine-induced toxicity on the liver and kidney in male albino Wistar rats was examined. To this effect, thirty-six (36) albino Wistar rats of weight 100-110 g were utilized and weight-matched animals placed in 6 groups of 6 rats each. After 30 days of the combined administration of codeine and the rice husk extract, the experimental animals were assayed for basic liver and renal markers such as AST, ALP, ALT, total protein, albumin, conjugated and total bilirubin, urea, creatinine, and electrolytes (sodium, potassium, chloride, and bicarbonate). Rice husks were collected from a local rice mill, and the extraction was done with methanol. *Findings*. Rice husk extract (RHE) significantly ameliorated the recorded hepatic damage. More so, the extract showed a significant action on the renal markers as well. A histopathology examination of the liver and kidney tissues revealed that RHE showed a hepatorenal ameliorative potential in a dose-dependent manner. *Conclusion*. Phytonutrient from RH possesses a healing ability against opioid-induced hepatorenal toxicity. Thus, RH is safe for human and may be adopted to obviate and manage codeine-induced hepatorenal damage or injury. *Significance and Novelty*. Data on the application of RHE as a phytonutrient to combat liver and kidney injuries were demonstrated. Future studies should evaluate its potential on other organs.

## 1. Introduction

The fact that the liver and kidney play a crucial part in drug metabolism explains why practically every medicine has been linked to hepatorenal toxicity. Hepatic metabolism is primarily a system that transforms substances into products that are more readily eliminated by the kidney and typically have a lesser pharmacologic action than the original compound [1]. Drug metabolites excreted by the kidneys may have higher activity and/or greater toxicity than the original drug, and they may also cause cellular damage that results in renal failure.

Codeine, a commonly used opioid, is useful as analgesic agent for the treatment of moderately severe acute or chronic pain [2]. Codeine has no significant analgesic effect; it is only through its bioactivation by CYP2D6 leading to morphine that it can impact its analgesic effect. It is converted in the liver by an enzyme cytochrome P450 enzyme (CYP) 2D6 to morphine which itself is an active substance and 2 to 4 times more potent than codeine [3]. Further, biotransformation results in inactive metabolites, which are excreted by kidneys.

In the field of pain management, there is increasing interest in the role of genetics on drug targets and metabolism [4]. Variations in the structure of genes are called genetic polymorphisms [5]. A gene that has been altered by a polymorphism is referred to as an allele of the original gene [5]. Polymorphisms can also influence the effects or actions of medications (pharmacodynamics (PD)) brought on by receptor binding properties of a particular drug, including adverse drug effects and efficacy [6]. Polymorphisms in the CYP450 enzymes have been found to have significant effects on drug metabolism. There is a spectrum of responses after the same dose of an opioid has been given to different individuals, ranging from no pain relief to toxicity, depending on whether the individual is a poor metaboliser (PM), extensive metabolisers (EM), or ultrarapid metabolisers (UM) [7].

The impact of polymorphisms of CYP450 enzymes on opioid effects can be seen in opioids that are commonly used in clinical practice. CYP450 enzymes have been found to be directly responsible for variability in the effects of codeine (CYP2D6), tramadol (CYP2D6), fentanyl (CYP3A5), and methadone (CYP2B6) [5]. It is important to note that the CYP450 system can also play a crucial role in transforming a drug that has no intrinsic active properties into a drug that does. When this occurs, the parent compound is referred to as a prodrug. Codeine is an example of a prodrug as it requires metabolism to morphine by the CYP450 system for it to have any opioid activity or analgesia [5, 8]. The National Survey on Drug Use and Health 2019 has confirmed the massive scale of drug problem. Over 15% of the adult population are into psychoactive drug substances [9]. While the widespread illicit drug use lingers, the survey also noted a major gap in health care system meeting the needs for treatment and care for individuals with drug abuse challenges. The abuse of prescription opioids such as codeine has reached an alarming rate currently in several countries.

The annual production of rice generates huge quantities of by-products such as the straw, bran, and husk. Currently, only about 20% of the by-products are used for practical purposes. However, most by-products are gotten rid of either through ground burying or via burning, thereby creating environmental pollution leading to greenhouse gas emission. Several studies have proven that bioactive compounds rich in natural products exhibit various therapeutic activities [10]. The various waste products, such as broken rice, rice bran, rice straw, and rice husk, can be fully utilized by food industries and pharmacological companies as their raw materials. In recent years, this by-product from different regions of the world has been ascertained to possess high phenolic compound [11]. As per Punvittayagul et al. [12], rice husk possesses antioxidants and polyphenolic compounds that shields the inner materials from oxidative damage. So many nutraceuticals derived from rice husk contain significant amount of phytochemicals, minerals, and vitamins with antioxidant activities. According to Jeon et al. [13], phenolic compounds from RHE have shown significant antioxidant proclivity against singlet oxygen and also inhibited hydrogen peroxide-induced damage to DNA in human lymphocytes. Kim et al. [14] also supported the data of Jeon et al. [13] that RHE showed effective antioxidant actions. Several research works have shown that protective layer of seeds contains strong antioxidants such as hydrocinnamic acid derivatives, phytic acid, vanillin, flavonoids, and syringaldehyde [15, 16]. Nilnumkhum et al. [17] suggested that the phenolic compounds specifically vanillic acid in purple rice husk extract (PRHE) can be effective as cancer chemopreventive agent and antimutagenic agent. Chung et al. [18] reported that methanolic extracts from rice hulls (MERH) could possess great antioxidant activity and chemopreventive bioactive properties against initiation stage of breast cancer. Punvittayagul et al. [12] reported that purple rice husk extract contains phytochemicals effective for suppression of hepatocyte proliferation, apoptosis stimulation, and detoxifying enzymes in the liver. Sankam et al. [19] also reported that PRHE contains significant amount of tocols that could exhibit strong inhibitory effect on hepatocarcinogenesis. According to Khat-Udomkiri et al. [20], xylooligosaccharides extracted from RH could serve as an improved source of nutraceutical for diabetes. Johar et al. [21] had effectively extracted cellulose fibre from RH with the aid of acid hydrolysis action. In response to these findings, we hypothesized that concurrent treatment with rice husk extract would reduce liver and kidney toxicities during exposure to codeine. This research established the ameliorative potential of RHE on liver and kidney damages in codeine-induced Wistar albino rats.

## 2. Materials and Methods

### 2.1. Procurement of Experimental Rats

A total of thirty-six (36) albino Wistar rats with weights between 100 and 110 g were used for this experiment. The rats were procured and housed in the Department of Pharmacology, University of Port Harcourt, Rivers State, Nigeria. The rats were left for 1 week to accustom to the laboratory conditions during which they were administered normal feed (Topfeeds grower mash) and clean water. The rats were handled according to the approved experimental protocol of the University of Port Harcourt Ethics Committee with Approval No. UPH/CEREMAD/REC/MM79/026. The animals were grouped into six groups comprising six rats per group.

### 2.2. Collection of Plant Sample

Rice husks were collected from a local rice mill at Awgu town, Enugu State, South East Nigeria, in December 2020. The husks were further ground, and the samples were filtered through a 48-mesh sieve. Rice husk powder was packed in plastic bags and taken to the laboratory. The powdered samples were kept in an airtight container till further use.

### 2.3. Preparation of Plant Extract

The powdered rice husk sample (100 g) was weighed and packed in the Whatman No. 1 filter paper. The packed sample was subjected to Soxhlet extractions with 400 ml (1 : 4 *w*/*v*) of methanol as the solvent. The extraction was allowed to run for about 5-9 hours (60 to 65 cycles) until a colourless liquid solution was obtained. The resultant extract was concentrated at 40-50°C using a rotary vacuum evaporator with an ultracryostat and further dried in an electrothermal oven. The brown paste solid obtained was stored in an airtight container in the refrigerator at 4°C till future use.

### 2.4. Experimental Design

The experimental rats were grouped into six groups of six rats each. The group I rats served as the normal control and were given only normal chow and clean water throughout the experimental period. The group II rats received only high dose of codeine at 10 mg/kg body weight of rats. The group III rats received 500 mg rice husk extract/kg body weight only. The group IV to VI rats were administered 10 mg codeine/kg b.w alongside rice husk extract at three different concentrations (250 mg, 500 mg, and 1,000 mg), respectively. After 30 days of treatment, the rats were sacrificed under exposure to light ether anaesthesia and blood collected via cardiac puncture for the liver and renal function analysis. The liver and kidney of the rats were also harvested for histopathology examination.

### 2.5. Method of Analysis

Biochemical assay examined on the liver includes alkaline phosphatase (ALP), aspartate aminotransferase (AST), alanine aminotransferase (ALT), total protein, albumin, and conjugated and total bilirubin, while urea, creatinine, and electrolytes (sodium, potassium, chloride, and bicarbonate) were assayed on the kidney. These assays were conducted with their individual diagnostic kits (Fortress Diagnostics, Antrim Technology, BT41 1QS, United Kingdom) according to the manufacturer's instructions.

### 2.6. Histopathological Examination

The liver and kidney tissues were cut into sections to moderate their thickness after being fixed in 10% formaldehyde to reduce bacteria load and tissue damage. Tissues were dehydrated starting with 50% alcohol for two hours, 70% alcohol for another two hours, 95% alcohol for twelve hours (overnight), and then absolute (100%) alcohol for two hours with mild agitation. After the sections have been made, the slides were stained using haematoxylin and eosin (H&E) stains. They are the most used combination of stains for routine histopathology examination. After staining, the sectioned tissues were prepared as a permanent preparation for microscopic examination by mounting the section in a suitable medium under a glass cover slip using a mountant. The slides were then viewed under a microscope at 400x magnification, and the photomicrograph was captured and interpreted accordingly.

### 2.7. Statistical Analysis

All numerical data were subjected to statistical analysis. The analysis of variance (ANOVA) of SPSS (version 25.0) was applied to the sequence of observations for the purpose of comparative analysis. Values were reported as mean ± standard error of mean (SEM), while Duncan's multiple comparisons were used to test for significant differences between the treatment groups. The results were considered significant at *p* values of less than 0.05 (*p* < 0.05), that is, at 95% confidence level.

## 3. Results

**Figure 1:**
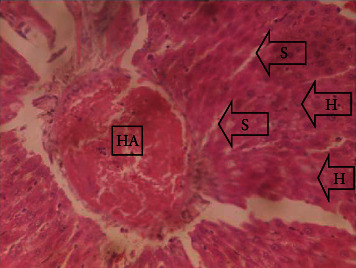
Photomicrographs of the normal liver of the group I rats showing intact hepatocytes (H), sinusoids (S) containing Kupffer cells, and congested hepatic artery (HA).

**Figure 2:**
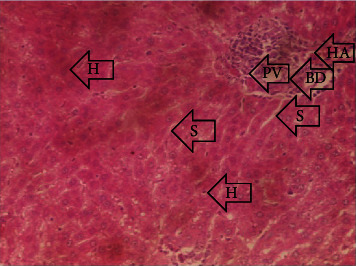
Photomicrographs of the group II rats showing distorted liver of periportal infiltration of inflammatory cells, patent portal vein (PV), hepatic artery (HA), and bile duct (BD), also with intact hepatocytes and sinusoids.

**Figure 3:**
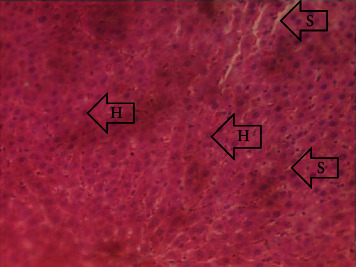
Photomicrographs of the group III rats showing normal liver of intact hepatocytes, sinusoids containing Kupffer cells, and congested capillaries.

**Figure 4:**
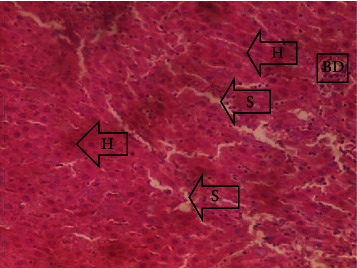
Photomicrographs of the group IV rats showing mildly distorted liver of sinusoids with capillaries and increased inflammatory cells, intact hepatocytes, patent bile duct (BD), and congested hepatic artery (HA).

**Figure 5:**
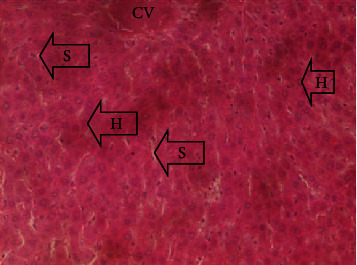
Photomicrographs of the group IV rats with normal liver of congested central vein (CV), intact hepatocytes, and sinusoids containing Kupffer cells.

**Figure 6:**
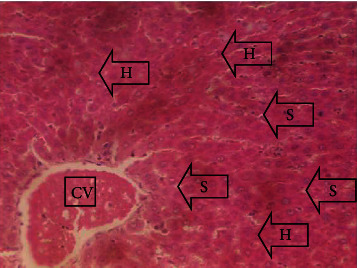
Photomicrographs of the group VI rats with normal liver of intact hepatocytes, sinusoids containing Kupffer cells, and congested central vein.

**Figure 7:**
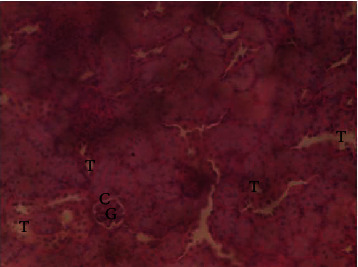
Photomicrographs of the group I animal showing normal kidney of glomeruli (G) containing mesangial cells, mesangial matrix, and capillaries, patent Bowman's capsule (C), and renal tubules (T) lined by simple epithelial cells.

**Figure 8:**
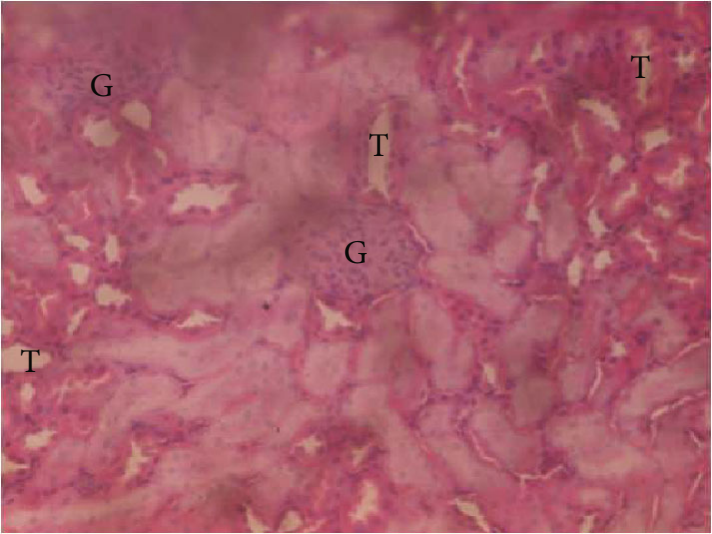
Photomicrographs of the group II rats showing normal kidney.

**Figure 9:**
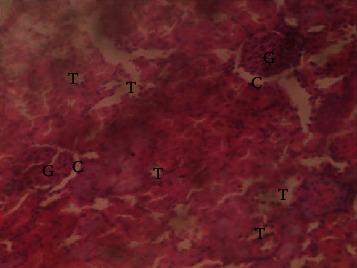
Photomicrographs of the group III rats with normal kidney of glomeruli containing mesangial cells, mesangial matrix, and capillaries, patent Bowman's capsule spaces, and intact renal tubules.

**Figure 10:**
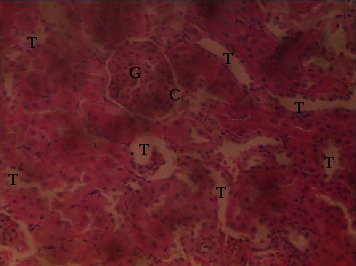
Photomicrographs of the group IV rats with normal kidney.

**Figure 11:**
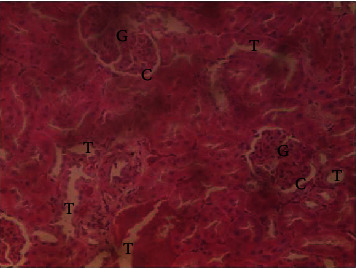
Photomicrographs of the group V rats showing normal kidney.

**Figure 12:**
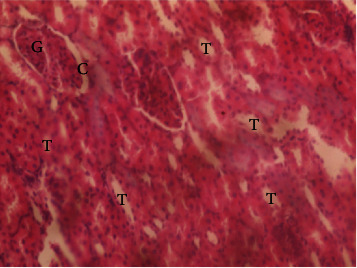
Photomicrographs of the group VI rats showing normal kidney.

The results of the liver and renal function assay recorded are presented in Tables 1 and 2, respectively. Referring to Table 1, the AST activity of the codeine control group was significantly higher (*p* < 0.05) than the rest of the groups with a value of 55.30 U/l. The normal control group recorded the least AST activity with 33.65 U/l. There was no significant difference in ALT activity among the groups, though the codeine control group recorded higher ALT activity than the other groups. More so, the ALP activity of the codeine control group was significantly higher (*p* < 0.05) than the rest of the groups. The total protein concentration of the RHE control group was significantly higher (*p* < 0.05) than that of the rest of the groups with the exception of the group that received codeine with 1,000 mg/kg RHE. The codeine control group recorded the least total protein concentration. The albumin concentration of the RHE control group was also significantly higher (*p* < 0.05) than that of the rest of the groups. The codeine control group also recorded the least albumin concentration. The total and conjugated bilirubin concentrations of the codeine control group were significantly higher (*p* < 0.05) than that of the rest of the groups, while the RHE control group recorded the least total and conjugated bilirubin concentrations, respectively. From the renal marker result recorded in the study and as referred to Table 2, the urea and creatinine concentrations of the codeine control group were significantly higher (*p* < 0.05) than that of the rest of the groups, while the RHE control group recorded the least concentrations of urea and creatinine, respectively. There was also a noticeable gradual decline of these markers with increase in RHE concentration. More so, the electrolyte level showed that sodium ion, potassium ion, chloride ion, and bicarbonate ion concentrations of the codeine control group were significantly higher (*p* < 0.05) than that of the rest of the groups, while the RHE control group recorded the least electrolyte concentrations. The decline in the electrolytes was also concentration dependent. The photomicrograph of the histopathology examination on the liver and kidney tissues is shown in Figures 1–12. From the photomicrograph of the hepatic tissues examined, the normal control showed a histological normal liver with intact hepatocytes (H), sinusoids (S) containing Kupffer cells, and congested hepatic artery (HA) (refer to Figure 1). The codeine control group (refer to Figure 2) showed a histological distorted liver with periportal infiltration of inflammatory cells, patent portal vein (PV), hepatic artery (HA), and bile duct (BD), though the hepatocytes and sinusoids remained intact. The RHE control group (refer to Figure 3) showed a histological normal liver with intact hepatocytes and sinusoids containing Kupffer cells and congested capillaries. The group that received both codeine and 250 mg/kg RHE (refer to Figure 4) showed a mildly distorted liver, with sinusoids, capillaries and increased inflammatory cells, intact hepatocytes, patent bile duct (BD), and congested hepatic artery. The group that received both codeine and 500 mg/kg RHE (refer to Figure 5) showed a histological normal liver with congested central vein (CV), intact hepatocytes, and sinusoids containing Kupffer cells. The group that received both codeine and 1,000 mg/kg RHE (refer to Figure 6) showed a histological normal liver with intact hepatocytes, sinusoids containing Kupffer cells, and congested central vein. From the photomicrograph of the renal tissues examined, the normal control showed a histological normal kidney with glomeruli (G) containing mesangial cells, mesangial matrix, and capillaries, patent Bowman's capsule (C), and renal tubules (T) lined by simple epithelial cells (refer to Figure 7). The codeine control group (Figure 8) showed a histological normal kidney. The RHE control group (refer to Figure 9) showed a histological normal kidney with glomeruli containing mesangial cells, mesangial matrix, and capillaries, patent Bowman's capsule spaces, and intact renal tubules. The groups that received both codeine and RHE at 250 mg/kg, 500 mg/kg, and 1,000 mg/kg (refer to Figures 10–12) showed histological normal kidneys, respectively.

The effect of RHE on hepatic tissues of codeine-administered male albino Wistar rats is described in the Figures 1–6.

The effect of RHE on renal tissues of codeine-administered male albino Wistar rats is described in the Figures 7–12.

## 4. Discussion

This study was conducted to examine the ameliorative effect of rice husk methanol extract against codeine-induced toxicity of the liver and kidney in animal model. Codeine was considered a narcotic agent due to its alarming misuse in Africa. The Federal Government of Nigeria has recently placed ban on the production and importation of codeine containing cough syrup [22]. Studies have reported the antioxidant and anti-inflammatory properties of rice husk [14, 16]. Data from present study revealed that rice husk extract was effective against liver and kidney toxicities in a dose-dependent manner. The highest ameliorative activity of RHE was recorded when administered at the highest concentration of 1000 mg/kg (refer to Tables 1 and 2). Despite wide use of codeine for years, limited records have been linked to elevated serum enzymes during clinical procedures, and there is also no evidence associating its high-dose intake to kidney and liver impairments and/or further inflammation of the organs. Hepatotoxicity induced by codeine intake is among the most commonly used model system for the screening of misuse of opioid drugs. As per Owoade et al. [23], codeine intake stimulated serum AST, ALT, and ALP elevation. This research revealed a significant rise in ALT, ALP, AST, and total and conjugated bilirubin and a significant decrease in albumin and total protein concentrations in the serum of the codeine control group. Salashoor et al. [24] posited that elevated liver enzymes could be linked to centrilobular necrosis and extreme degeneration of the liver. Thus, elevated liver enzymes recorded may reflect hepatic damage. According to Nnadiukwu et al. [25], elevated serum ALT is an indication of liver disease. Elevated ALP activity recorded by the codeine control group may be considered a sensitive marker of early stage of cholestasis [25]. ALT or AST is a liver-specific enzyme, and its elevated concentrations are usually linked to lot of health problems. Bilirubin is a metabolic product of haemoglobin breakdown which undergoes conjugation with glucuronic acid in hepatocytes to increase its water solubility. According to El-Gizawy et al. [26], bilirubin determination is used to measure hepatic function, necrosis severity, conjugation, and excretory efficiency of hepatocytes, and any abnormal increase in bilirubin levels in the serum denotes hepatobiliary illness and severe hepatocellular dysfunction. Interestingly, the recorded increased levels of these enzymes and bilirubins were decreased in the group treated with RHE. The histopathological study of the liver (refer to Figures 1–6) also revealed that high-dose codeine administration contributed to some histological distortion and inflammation of the hepatocytes. However, this was not the case with the normal control group alongside the treated groups except for the group that received RHE at 500 mg/kg that showed a histological normal liver with congested central vein. This implied that the RHE prevented the liver damage which was confirmed by the decreased concentration of the hepatic markers and reduced histopathological injury of the hepatic tissues among the treated groups. This action of RHE may be attributed to the presence of terpenoid which has the ability to effectively ease liver-related injuries and/or diseases. According to Gong et al. [27], plant-derived terpenoids can effectively alleviate liver fibrosis and liver injury-related diseases. Andro, a plant-derived terpenoid, could lower ALT and hepatic total cholesterol levels and reduces the expression of NLRP3 (NLR family pyrin domain containing 3) and related inflammatory factors by increasing antioxidant and anti-inflammatory activities in nonalcoholic fatty liver disease (NAFLD) mice [28]. Meanwhile, decreased total protein and albumin concentration recorded by the codeine-administered group may be linked to the reduction in hepatocytes manifested by variation on the hepatic cell membrane which in reverse may lead to low hepatic proclivity to synthesize protein and albumin [29]. These decreased total protein and albumin were enhanced by the RHE suggesting increased healing capacity of the extract on the liver. According to Okokon et al. [29], elevated total protein and albumin indicate the restoration of endoplasmic reticulum and ameliorative action that synthesizes protein. Kidney markers such as urea, creatinine, and electrolytes were assayed to estimate renal toxicity. According to McCann et al. [30], urea assay in clinical studies is crucial in estimating amino acid metabolism, its elimination via urinary excretion and nephrotoxicity of xenobiotics, whereas plasma creatinine is adopted to measure glomerular filtration rate and renal function [31]. The present study recorded significant increased urea and creatinine levels in the codeine-administered group than the rest of the groups. This can be reported as an evidence of renal injury, which may lead to impaired kidney function. Owoade et al. [23] recorded an increase in urea and creatinine concentrations in tramadol-administered rats. However, treatment with RHE in doses of 250 mg, 500 mg, and 1000 mg, respectively, significantly reduced the high urea and creatinine concentrations. According to Ishimoto et al. [32], the kidney is crucial in maintaining stable electrolyte concentrations in the blood irrespective of physiological body adjustment. The study recorded elevated plasma electrolytes (sodium, potassium, chloride, and bicarbonate) in the codeine-administered rats which were restored to normal after RHE administration. The histopathological study of the kidney showed no obvious change in the codeine-administered groups (refer to Figure 8), while the groups cotreated with codeine and RHE (refer to Figures 10–12) showed intact renal tubule, patent Bowman's capsular space, and glomeruli containing mesangial cells, mesangial matrix, and capillaries after the exposure period of 30 days. According to Monir et al. [33], reactive oxygen species have been reported as the hallmark mechanism for the development of kidney injury/damage via increased kidney biomarkers. Natural compounds that possess high antioxidant and anti-inflammatory effects are expected to possess a renal protective effect [34]. Several studies have reported the antioxidant effect of vitamin E against kidney injury/damage. Vitamin E was able to bind to superoxide free radicals and prevent damage caused by reactive oxidant species [35]. RHE contains significant amount of vitamin E and other vitamins. A meta-analysis by Cho et al. [36] reported that vitamins and analogues are effective in the prevention of kidney injury/damage. The improvement in the kidney biomarkers seen in the RHE-treated groups explains the enhanced kidney histology.

## 5. Conclusion

This study affirmed that high-dose codeine intake could lead to alteration in the biochemical indices of the liver and kidney as well as organ damage. The study also revealed that RHE contains vitamins and phytochemicals with antioxidant and anti-inflammatory properties against codeine-induced hepatorenal organ damage in rats. This research offers valuable facts on rice husk extract for application in alternative medicine and thus calls for further investigation and isolation of bioactive compounds of RHE.

## Figures and Tables

**Table 1:** Effect of rice husk extract on liver markers in codeine-administered male albino Wistar rats.

Parameter	NC	COD. only	RHE only	COD.+250 mg RHE	COD.+500 mg RHE	COD.+1,000 mg RHE
AST (U/l)	33.65 ± 20.46^a^	55.30 ± 8.41^b^	40.86 ± 1.77^ab^	46.31 ± 7.66^ab^	45.15 ± 5.27^ab^	44.01 ± 6.52^ab^
ALT (U/l)	8.79 ± 5.47^a^	13.15 ± 1.98^a^	9.82 ± 0.62^a^	12.40 ± 2.65^a^	11.65 ± 3.54^a^	10.51 ± 1.24^a^
ALP (U/l)	20.45 ± 10.42^abc^	48.48 ± 12.92^c^	13.64 ± 14.20^a^	47.73 ± 2.28^bc^	35.61 ± 7.31^abc^	18.94 ± 28.87^ab^
Total protein (g/dl)	56.33 ± 2.08^bc^	46.33 ± 4.04^a^	63.00 ± 2.00^d^	55.00 ± 2.00^b^	56.67 ± 1.53^bc^	60.00 ± 2.00^cd^
Albumin (g/dl)	33.00 ± 2.00^b^	26.67 ± 1.53^a^	43.67 ± 3.06^d^	31.67 ± 1.53^b^	35.33 ± 1.53^bc^	37.33 ± 3.06^c^
Total bilirubin (*μ*mol/l)	6.30 ± 0.56^b^	17.20 ± 1.35^d^	4.33 ± 0.35^a^	12.13 ± 1.00^c^	7.60 ± 0.62^b^	7.20 ± 0.40^b^
Conjugated bilirubin (*μ*mol/l)	3.93 ± 0.67^b^	11.10 ± 0.96^d^	2.77 ± 0.25^a^	6.87 ± 0.60^c^	4.53 ± 0.15^b^	4.57 ± 0.47^b^

Groups with different superscripts are significantly different at *p* < 0.05, while groups with same superscripts are not. Key: NC = normal control; COD. only = administered 10 mg codeine/kg b.w of rat; RHE only = administered 500 mg of rice husk extract/kg b.w of rats; COD.+250 mg RHE = administered 10 mg codeine/kg b.w of rats +250 mg rice husk extract; COD.+500 mg RHE = administered 10 mg codeine/kg b.w of rats +500 mg rice husk extract; COD.+1,000 mg RHE = administered 10 mg codeine/kg b.w of rats +1000 mg rice husk extract.

**Table 2:** Effect of rice husk extract on renal markers in codeine-administered male albino Wistar rats.

Parameter	NC	COD. only	RHE only	COD.+250 mg RHE	COD.+500 mg RHE	COD.+1,000 mg RHE
Urea (mmol/l)	4.90 ± 0.26^a^	7.23 ± 0.31^d^	4.70 ± 0.20^a^	6.43 ± 0.55^c^	5.60 ± 0.26^b^	5.03 ± 0.15^a^
Creatinine (*μ*mol/l)	105.33 ± 3.51^a^	146.67 ± 9.71^c^	105.67 ± 3.51^a^	136.67 ± 4.16^c^	124.33 ± 8.14^b^	106.67 ± 3.51^a^
Sodium (mmol/l)	144.33 ± 8.08^b^	186.00 ± 9.64^c^	118.00 ± 4.58^a^	166.67 ± 29.37^bc^	161.67 ± 11.06^bc^	142.33 ± 4.16^ab^
Potassium (mmol/l)	4.60 ± 0.26^ab^	6.47 ± 0.70^c^	3.73 ± 0.15^a^	5.17 ± 1.11^b^	5.30 ± 0.44^b^	4.47 ± 0.25^ab^
Chloride (mmol/l)	63.60 ± 3.00^c^	66.00 ± 3.61^c^	51.33 ± 3.06^a^	64.33 ± 1.53^c^	56.33 ± 2.08^b^	53.67 ± 1.53^ab^
Bicarbonate (mmol/l)	24.00 ± 2.00^a^	32.00 ± 2.00^c^	23.67 ± 1.53^a^	28.40 ± 2.00^b^	26.33 ± 1.53^ab^	26.33 ± 2.08^ab^

Groups with different superscripts are significantly different at *p* < 0.05, while groups with same superscripts are not. Key: NC = normal control; COD. only = administered 10 mg codeine/kg b.w of rat; RHE only = administered 500 mg of rice husk extract/kg b.w of rats; COD.+250 mg RHE = administered 10 mg codeine/kg b.w of rats +250 mg rice husk extract; COD.+500 mg RHE = administered 10 mg codeine/kg b.w of rats +500 mg rice husk extract; COD.+1,000 mg RHE = administered 10 mg codeine/kg b.w of rats +1000 mg rice husk extract.

## Data Availability

The datasets used and/or analyzed during the current study are available from the corresponding author upon request.

## References

[1] Elkhateeb A., El Khishin I., Megahed O., Mazen F. (2015). Effect of *Nigella sativa Linn* oil on tramadol-induced hepato- and nephrotoxicity in adult male albino rats. *Toxicology Reports*.

[2] Ćelić I., Bach-Rojecky L., Merćep I., Soldo A., Petrak A. K., Bučan A. (2020). Resolving issues about efficacy and safety of low-dose codeine in combination analgesic drugs: a systematic review. *Pain and Therapy*.

[3] Ballester P., Muriel J., Peiró A. M. (2022). CYP2D6 phenotypes and opioid metabolism: the path to personalized analgesia. *Expert Opinion on Drug Metabolism & Toxicology*.

[4] Ting S., Schug S. (2016). The pharmacogenomics of pain management: prospects for personalized medicine. *Journal of Pain Research*.

[5] Argoff C. E. (2014). An introduction to pharmacogenetics in pain management: knowledge of how pharmacogenomics may affect clinical care. *Practical Management of Pain*.

[6] Gudin J. (2012). Opioid therapies and cytochrome P450 interactions. *Journal of Pain Symptom Manage*.

[7] Webster L. R., Belfer I. (2016). Pharmacogenetics and personalized medicine in pain management. *Clinics in Laboratory Medicine*.

[8] Johnson M. I., Radford H. (2016). CYP2D6 polymorphisms and response to codeine and tramadol. *Analgesia & Resuscitation : Current Research*.

[9] Salolainen I., Oksanem A., Kaakinen M., Sirola A., Peak H. J. (2020). The role of perceived loneliness in youth addictive behaviors: cross-national survey study. *JMIR Mental Health*.

[10] Pandey K. B., Rizvi S. I. (2009). Plant polyphenols as dietary antioxidants in human health and disease. *Oxidative Medicine and Cellular Longevity*.

[11] Sung W., Stone M., Sun F. (2007). Analysis of volatile constituents of different temperature rice hulls liquid smoke. *Chia-Nan Annual Bulletin*.

[12] Punvittayagul C., Chariyakornkul A., Sankam P., Wongpoomchai R. (2021). Inhibitory effect of Thai purple rice husk extract on chemically induced carcinogenesis in rats. *BMC Complementary and Alternative Medicine*.

[13] Jeon K., Park E., Park H., Jeon Y., Cha S., Lee S. (2006). Antioxidant activity of far-infrared radiated rice hull extracts on reactive oxygen species scavenging and oxidative DNA damage in human lymphocytes. *Journal of Medicinal Food*.

[14] Kim S. P., Yang J. Y., Kang M. Y., Park J. C., Nam S. H., Friedman M. (2011). Composition of liquid rice hull smoke and anti-inflammatory effects in mice. *Journal of Agricultural and Food Chemistry*.

[15] Lee S. C., Kim J. H., Jeong S. M. (2003). Effect of far-infrared radiation on the antioxidant activity of rice hulls. *Journal of Agricultural and Food Chemistry*.

[16] Butsat S., Siriamornpun S. (2010). Phenolic acids and antioxidant activities in husk of different Thai rice varieties. *Food Science and Technology International*.

[17] Nilnumkhum A., Punvittayagul C., Chariyakornkul A., Wongpoomchai R. (2017). Effects of hydrophilic compounds in purple rice husk on AFB1-induced mutagenesis. *Molecular & Cellular Toxicology*.

[18] Chung N. J., Choi K. C., Lee S. A., Baek J. A., Lee J. C. (2015). Rice hull extracts inhibit proliferation of MCF-7 cells with G1 cell cycle arrest in parallel with their antioxidant activity. *Journal of Medicinal Food*.

[19] Sankam P., Punvittayagul C., Sringam K., Chaiyasut C., Wongpoomchai R. (2013). Antimutagenicity and anticlastogenicity of glutinous purple rice hull using in vitro and in vivo testing systems. *Molecular & Cellular Toxicology*.

[20] Khat-Udomkiri N., Toejing P., Sirilun S., Chaiyasut C., Lailerd N. (2020). Antihyperglycemic effect of rice husk derived xylooligosaccharides in high-fat diet and low-dose streptozotocin-induced type 2 diabetic rat model. *Food Science & Nutrition*.

[21] Johar N., Ahmad I., Dufresne A. (2012). Extraction, preparation and characterization of cellulose fibres and nanocrystals from rice husk. *Industrial Crops and Products*.

[22] Ezenwa M. O., Orjiakor T. C., Ukwuma M. C., Oraetue H. I., Ude E. N., Nweze T. (2019). Tracking opiate routes in Nigeria: identifying trafficking routes through dealers and users of tramadol and codeine. *Drugs in the Nigerian Population*.

[23] Owoade A. O., Abdullateef A., Adetutu A., Adetutu A., Olorunnisola O. S. (2019). Codeine-mediated haematoxicity, hepatotoxicity and nephrotoxicity in male albino rats. *Asian Journal of Research in Medical and Pharmaceutical Sciences*.

[24] Salashoor M. R., Roshankhan S., Hosseni P., Jalili C. (2018). Genistein improves liver damage in male mice exposed to morphine. *Chinese Medical Journal*.

[25] Nnadiukwu T. A., Monago C. C., Chuku L. C. (2017). Synergistic effect of ethanol extracts of moringa oleifera and pleurotus ostreatus on liver enzymes and some renal functions of alloxan-induced diabetic wistar albino rats. *International Journal of Biochemistry Research & Review*.

[26] El-Gizawy M. M., Hosny E. N., Mourad H. H., Razik A. E., Amira N. (2020). Curcumin nanoparticles ameliorate hepatotoxicity and nephrotoxicity induced by cisplatin in rats. *Naunyn-Schmiedeberg’s Archives of Pharmacology*.

[27] Gong J., Yang F., Yang Q. (2020). Sweroside ameliorated carbon tetrachloride (CCl4)-induced liver fibrosis through FXR-miR-29a signaling pathway. *Journal of Natural Medicines*.

[28] Liu Y. T., Chen H. W., Lii C. K. (2020). A diterpenoid, 14-deoxy-11, 12-didehydroandrographolide, in andrographis paniculata reduces steatohepatitis and liver injury in mice fed a high-fat and high-cholesterol diet. *Nutrients*.

[29] Okokon J. E., Nyong G. E., Anyanwu B. C., Dick G. F. (2017). In vivo Zea mays inhibitory activities of husk extract and fractions of on alpha amylase and alpha glucosidase. *International Journal of Herbal Medicine*.

[30] McCann M. R., George De la Rosa M. V., Rosania G. R., Stringer K. A. (2021). L-carnitine and acylcarnitines: mitochondrial biomarkers for precision medicine. *Metabolites*.

[31] Pottel H., Björk J., Courbebaisse M. (2021). Development and validation of a modified full age spectrum creatinine-based equation to estimate glomerular filtration rate: a cross-sectional analysis of pooled data. *Annals of Internal Medicine*.

[32] Ishimoto Y., Tanaka T., Yoshida Y., Inagi R. (2018). Physiological and pathophysiological role of reactive oxygen species and reactive nitrogen species in the kidney. *Clinical and Experimental Pharmacology & Physiology*.

[33] Monir N., Saber M. M., Awad A. S., Elsherbiny M. E., Zaki H. F. (2022). Repression of inflammatory pathways with Boswellia for alleviation of liver injury after renal ischemia reperfusion. *Life Sciences*.

[34] Fang C. Y., Lou D. Y., Zhou L. Q. (2021). Natural products: potential treatments for cisplatin-induced nephrotoxicity. *Acta Pharmacologica Sinica*.

[35] Liu P., Feng Y., Wang Y., Zhou Y., Zhao L. (2015). Protective effect of vitamin E against acute kidney injury. *Bio-Medical Materials and Engineering*.

[36] Cho M. H., Kim S. N., Park H. W., Chung S., Kim K. S. (2017). Could vitamin E prevent contrast-induced acute kidney injury? A systematic review and meta-analysis. *Journal of Korean Medical Science*.

